# The valosin-containing protein is a novel mediator of mitochondrial respiration and cell survival in the heart *in vivo*

**DOI:** 10.1038/srep46324

**Published:** 2017-04-20

**Authors:** Paulo Lizano, Eman Rashed, Shaunrick Stoll, Ning Zhou, Hairuo Wen, Tristan T. Hays, Gangjian Qin, Lai-Hua Xie, Christophe Depre, Hongyu Qiu

**Affiliations:** 1 Department of Cell Biology and Molecular Medicine; New Jersey Medical School, Rutgers, The State University of New Jersey, Newark, NJ, 07103, USA; 2 Division of Physiology, Department of Basic Science, School of Medicine, Loma Linda University, Loma Linda, CA, 92324, USA; 3 Department of Biomedical Engineering, University of Alabama at Birmingham (UAB),Birmingham, AL, 35294, USA

## Abstract

The valosin-containing protein (VCP) participates in signaling pathways essential for cell homeostasis in multiple tissues, however, its function in the heart *in vivo* remains unknown. Here we offer the first description of the expression, function and mechanism of action of VCP in the mammalian heart *in vivo* in both normal and stress conditions. By using a transgenic (TG) mouse with cardiac-specific overexpression (3.5-fold) of VCP, we demonstrate that VCP is a new and powerful mediator of cardiac protection against cell death *in vivo*, as evidenced by a 50% reduction of infarct size after ischemia/reperfusion versus wild type. We also identify a novel role of VCP in preserving mitochondrial respiration and in preventing the opening of mitochondrial permeability transition pore in cardiac myocytes under stress. In particular, by genetic deletion of inducible isoform of nitric oxide synthase (iNOS) from VCP TG mouse and by pharmacological inhibition of iNOS in isolated cardiac myocytes, we reveal that an increase of expression and activity of iNOS in cardiomyocytes by VCP is an essential mechanistic link of VCP-mediated preservation of mitochondrial function. These data together demonstrate that VCP may represent a novel therapeutic avenue for the prevention of myocardial ischemia.

Acute myocardial ischemia (MI) and coronary artery disease remain among the top ranking causes of death and disability worldwide^1^. Mitochondrial dysfunction contributes to cell damage during ischemia/reperfusion (IR) and is central to cardiomyocyte death^2^,^3^,^4^. It has been shown that ischemic preconditioning (IPC), the gold standard for cardioprotection, attenuated the decline of mitochondrial function induced by ischemic injury, including oxidative phosphorylation, respiratory chain coupling and mitochondrial efficiency^5^,^6^,^7^,^8^,^9^. There is also increasing evidence that the mitochondrial permeability transition pore (mPTP) opening plays a central role in mediating both the necrotic and apoptotic components of IR injury, particularly at the onset of reperfusion, and inhibition of mPTP opening is considered to be a critical target of cardioprotection by IPC^3^,^10^,^11^,^12^,^13^,^14^.

We identified previously that the valosin-containing protein (VCP), a member of a family that includes ATP-binding proteins, promotes a significant reduction in apoptosis in isolated cardiomyocytes under cell stress^15^. These data support the hypothesis that over-expression of VCP *in vivo* in the heart may provide protection against ischemic injury.

To test such hypothesis, we generated a transgenic (TG) mouse with cardiac-specific overexpression of VCP. Our results demonstrate that overexpression of VCP significantly reduced the infarct size (IS) by 50% after IR compared to wild type (WT) mice. We also showed that VCP exhibited a new role on activation of mitochondrial respiration efficiency and inhibition of the mPTP opening. Using both genetic and pharmacological approaches, we also show that the effect of VCP on the cardiac mitochondrial function was dependent upon the increase of inducible isoform of nitric oxide synthase (iNOS) conferred by VCP.

## Results

### Cardiac specific overexpression of VCP in a TG mouse did not alter cardiac structure and function at baseline

**Figure 1:**
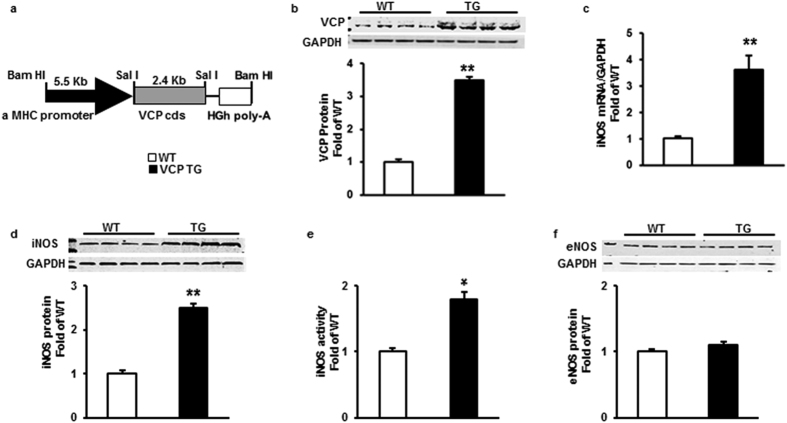
Cardiac-specific overexpression of VCP increases iNOS expression and activity in TG mouse hearts compared to WT. (**a**) VCP TG mouse construct. (**b**) Representative immunoblots and protein level of VCP overexpression in mouse hearts, n = 8 per group. (**c**) mRNA level of iNOS in mouse hearts by qPCR, n = 5 per group. (**d**) Representative immunoblots and protein level of iNOS in mouse hearts, n = 8 per group. (**e**) iNOS activity in mouse heart tissues, n = 5 per group. (**f**) Protein level of eNOS in mouse hearts, n = 4 per group. GAPDH was used as loading control. *p < 0.05.**p < 0.01 vs. WT. Data are the mean ± SEM.

The construct used to generate the TG mouse is shown in Fig. 1a. Different levels of VCP overexpression (from 3.5-fold to 10-fold) were found among the mice lines. In accordance with our previous data *in vitro*^15^, cardiac-specific overexpression of VCP dose-dependently increased iNOS expression in the hearts (from 2.5-fold to 10-fold, data not shown). Further characterization was performed on the VCP TG line with a 3.5-fold overexpression (Fig. 1b), since it increased iNOS expression by 2.5-fold in protein level when compared to WT (p < 0.01 vs. WT, Fig. 1d), which best reproduces the range of iNOS expression found in the second window of preconditioning (SWOP)^16^,^17^. Overexpression of VCP in this mouse line also increased the mRNA level of iNOS by 3.5 fold and the activity of iNOS by 1.8-fold compared to WT (p < 0.05 vs. WT, Fig. 1c and e). There was no change in eNOS expression in TG mouse heart compared to WT (Fig. 1f).

Compared to WT mice, the VCP TG mouse exhibited similar left ventricle (LV) weight-to-tibia length ratio (LV/TL), LV-to-body weight ratio (LV/BW), and the lung weight-to-tibia length ratio (LW/TL) in basal conditions (Table 1). VCP overexpression had no significant effect on myocyte cross-sectional area, collagen accumulation, and apoptosis (Table 1). No difference on structural and functional echocardiographic parameters was observed in VCP TG mice when compared to WT in terms of heart rate, LV end-diastolic diameter (LVEDD) and LV end-systolic diameter (LVESD), wall thickness, LV ejection fraction (LVEF) and fractional shortening (FS) (Table 1).

### Overexpression of VCP in a TG mouse model reduces IS during IR

**Figure 2:**
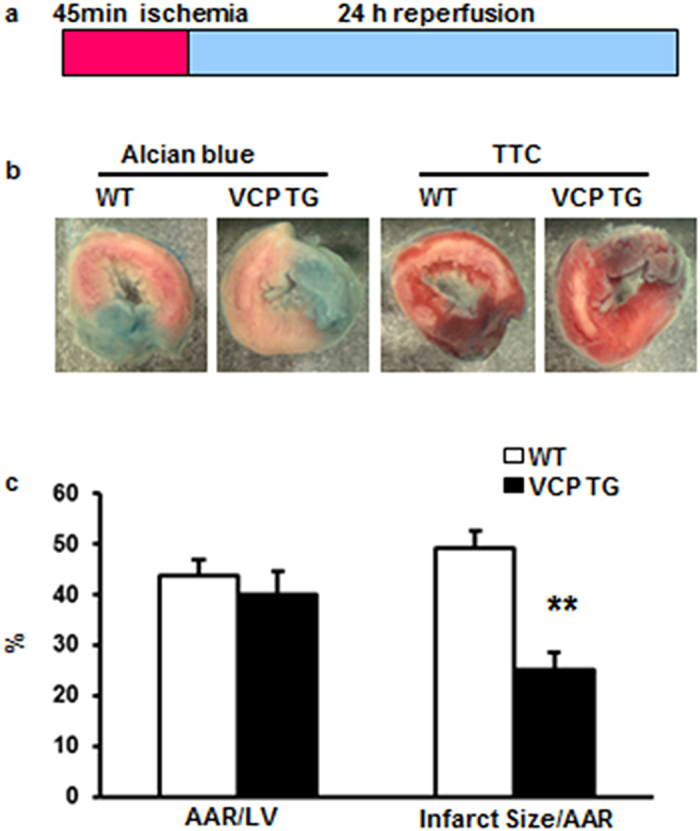
Overexpression of VCP reduces myocardial infarct size in TG mice. (**a**) Schema of the ischemia/reperfusion protocol. (**b)** Representative staining of left ventricle by Alcian blue and Triphenyl Tetrazolium Chloride (TTC). (**c**) Quantitation of AAR/LV and IS/AAR in WT and TG mouse hearts (n = 5 for TG and 7 for WT group). Data are the mean ± SEM. **p = 0.0006 vs. WT.

To test whether VCP is cardioprotective *in vivo*, 3 to 4-month old WT and TG mice were submitted to a 45 min ischemia followed by a 24 hours reperfusion as described in the Methods and illustrated in Fig. 2a. Although the area-at-risk (AAR) was similar, there was a 50% reduction of IS in TG mice compared with WT (p = 0.006 vs. WT, Fig. 2b and c), providing a cardioprotection against IR that is comparable to that observed in SWOP^16^,^17^.

### Mitochondrial distribution of VCP and iNOS was increased in VCP TG hearts

**Figure 3:**
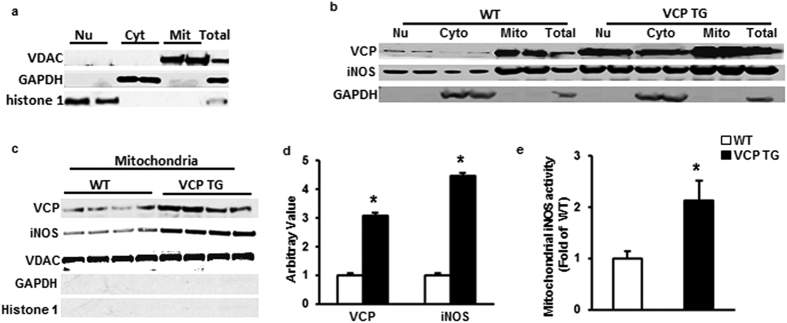
Sub-cellular distribution of VCP and iNOS in mouse heart. (**a**) Specific markers show the purity of the subcellular fractions: voltage-dependent anion channel (VDAC) for mitochondria (Mit); glyceraldehyde 3-phosphate dehydrogenase (GAPDH) for cytosol (Cyto), histone 1 for nucleus (Nu). (**b**) Representative subcellular distribution of VCP and iNOS in WT and VCP TG mouse hearts. GAPDH was used as a marker to exclude the contamination of cytosal in mitochondrial fraction. (**c**) Immunoblot of the expression of VCP and iNOS in mitochondrial fraction in TG and WT mouse hearts. VDAC was used as loading control of mitochondria. Negative staining of GAPDH and histone1 confirm the purity of mitochondrial fractions. (**d**) Ratio of VCP/VDAC and iNOS/VDAC in mitochondria in VCP TG mice compared to WT. n = 7/group. *p < 0.01 vs WT. (**e**) iNOS activity in isolated mitochondria from mouse hearts. n = 5/group. *p = 0.02 vs WT. Data are the mean ± SEM.

Our previous study showed that not only the increase of total endogenous iNOS expression but also its subsequent translocation to the mitochondria in cardiac myocytes is crucial for its effects on both mitochondrial respiration and cardioprotection^18^. To further test this concept in VCP TG mouse, subcellular fractions were extracted from both VCP TG and WT mouse hearts as described in the Methods. To determine the cellular distribution of iNOS as accurately as possible, the purity of each cellar fraction was confirmed by specific protein markers (Fig. 3a). As shown in Fig. 3b, VCP and iNOS exhibited a similar subcellular distribution in which both VCP and iNOS were localized primarily in the mitochondria in both WT and TG mouse hearts. Compared with WT, TG mice showed a 3.1-fold increase in VCP and 4.5-fold increase in iNOS in the mitochondrial fraction (P < 0.01 vs. WT, Fig. 3c and d). iNOS activity in isolated mitochondria from VCP TG mouse hearts was also increased by 2.1-fold compared to WT mice, indicating its functional activity in heart mitochondria in VCP TG mice (Fig. 3e).

### ADP-dependent oxygen consumption was enhanced in VCP in TG mouse heart

**Figure 4:**
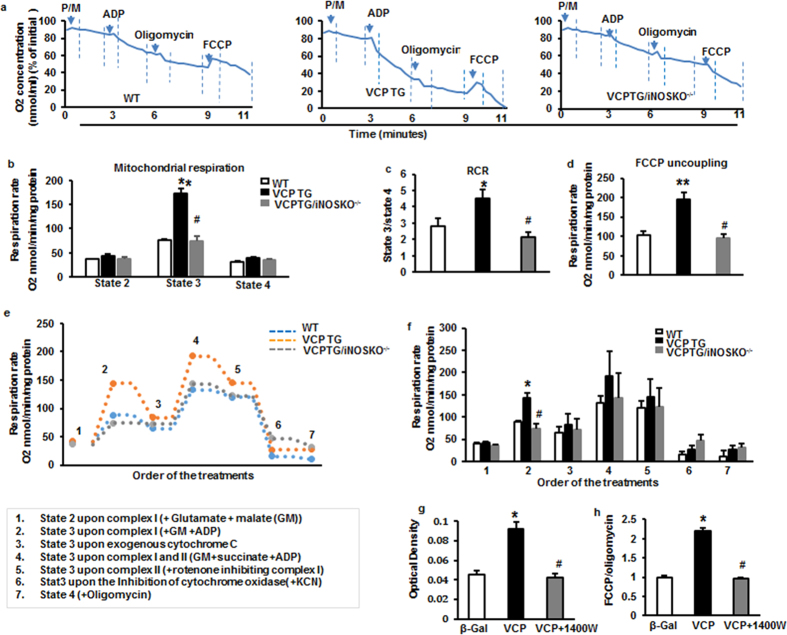
Effects of VCP overexpression on mitochondrial respiration. **(a**) An representative plot of relative oxygen concentration from each group (% of initial oxygen concentration nmol/ml). (**b)** Measurement of mitochondrial respiration rate (states 2 to 4) of mouse heart tissue measured by Clark electrode. State 2 and state 4 represent the substrate-dependent respiration rate measured under the addition of pyruvate and malate in the absence of exogenous ADP; State 3 represents ADP-stimulated respiration rate measured under the addition of ADP; (**c**) Efficiency of mitochondrial respiration as measured by the respiratory control ratio (RCR: State3/State4). (**d**) Maximum respiration capacity as measured after the addition of the uncoupler FCCP. n = 6 per group for b to d. *p < 0.05, **p < 0.01 vs. WT; ^#^p < 0.01 vs. VCP TG. (**e)** Representative tracing of oxygen consumption rate of isolated mitochondria from mouse hearts during a stepwise protocol (plots 1 to 7 indicating the order of treatment as 1. glutamate/malate, 2. ADP, 3. cytochrome c, 4. succinate, 5. rotenone, 6. inhibitor of cytochrome oxidase, KCN and 7. oligomycin). (**f**) Average oxygen consumption rates of panel e. n = 4–5 per group *p < 0.01 vs. WT; ^#^p < 0.01 vs. VCP TG. (**g**) iNOS activity and (**h**) Mitochondrial oxygen consumption rate (OCR) in RNCMs with or without iNOS inhibitor, 1400 W. n = 6/group for g to h. *p < 0.05 vs. Ad-ß-Gal; ^#^p < 0.05 vs. Ad-VCP. Data are the mean ± SEM.

Since the majority of VCP is localized in mitochondria, we tested the effect of VCP on mitochondrial respiration in the heart. We first tested the complex I-dependent mitochondrial respiration by measuring oxygen consumption in the presence of pyruvate and malate, two Complex I substrates, with and without ADP, as illustrated in Fig. 4a. Respiration rates of states 2 to 4 were determined by the oxygen consumption per minute normalized by mitochondrial proteins. As shown in Fig. 4b, although there was a modest increase in state 2 respiration rate in the mitochondria from VCPTG mice vs WT, the difference did not reach statistical significance. Mitochondria from VCP TG mouse heart tissues showed a significant increase in oxygen consumption at the ADP-dependent state 3 under complex I stimulation compared to WT, while no significant difference was seen in oxygen consumption states 4 (upon the addition of oligomycin, a known inhibitor of the ATP synthase) (Fig. 4b). The efficiency of mitochondrial respiration, as measured by the respiratory control ratio (RCR: state 3/state 4) was significantly increased in VCP TG mice versus WT (Fig. 4c). In addition, VCP TG mice also exhibited a significant increase in maximum respiration capacity versus WT as measured after the addition of the uncoupling agent carbonyl cyanide 4-trifluoro methoxyphenylhydrazone (FCCP) (Fig. 4d).

In addition, we tested the effect of VCP on complex II dependent respiration in the isolated mitochondria by adding succinate, a known Complex II substrate, in the presence of ADP, with and without the Complex I inhibitor rotenone. As shown in Fig. 4e and f, there was a remarkable increase in respiration rate from both WT and VCP TG mice after the addition of succinate compared to Complex I alone, suggesting that respiration under Complex II was additive. However, the difference of respiration rates between VCP TG and WT observed under stimulation of Complex I (Plot 2 in Fig. 4e and f) lost significance upon the simulation of Complex I and II after the addition of succinate (Plot 4 in Fig. 4e and f). We tested Complex II-specific respiration by adding the Complex I inhibitor, rotenone, and found that there was no longer a difference in respiration rate between VCP TG and WT (Plot 5 in Fig. 4e and f). These data together suggest that VCP may be predominantly affecting Complex I dependent respiration in VCP TG mice.

To determine whether the increase of oxygen consumption in VCP TG mice was the result of enhanced mitochondrial respiration, we used Cytochrome C to test the integrity of the mitochondrial membrane. As shown in Plot 3 of Fig. 4e and f, addition of cytochrome C did not increase the respiration rates of either group’s samples, indicating a high membrane integrity. Next, we added potassium cyanide (KCN), a known inhibitor of cytochrome oxidase. Our data showed that addition of KCN inhibited the respiration rates for both WT and VCP TG in a similar manner, indicating a direct effect on mitochondrial respiratory chain (Plot 6 in Fig. 4e and f). Moreover, there were no significant difference observed between VCP TG and WT upon the addition of oligomycin after the inhibition of cytochrome oxidase by KCN (Plot 7 in Fig. 4e and f).

### Genetic deletion of iNOS abolished VCP-mediated increase in mitochondrial respiration *in vivo*

We have shown that VCP dose-dependently increases the expression of iNOS in myocytes^15^. However, it is unknown if iNOS mediates the effect of VCP on mitochondrial respiration. To further determine whether the stimulation of mitochondrial respiration observed in VCP TG mouse heart is mediated by iNOS *in vivo*, a bigenic VCP TG/iNOS KO^−/−^ mouse was generated, in which iNOS was deleted from VCP TG mice (see Methods for mating strategy). Oxygen consumption was measured in these VCP TG/iNOS KO^−/−^ mice and compared with their litter-matched VCP TG/iNOS^+/+^ mice and WT/iNOS^+/+^ counterparts. As shown in Fig. 4a to c, the increase in mitochondrial respiration state 3 and in RCR observed in VCP TG mouse heart was abolished upon deletion of iNOS. There was also a significant decrease in maximum respiration capacity as measured after the addition of the uncoupling agent FCCP in VCP TG/iNOS KO^−/−^ mice compared to VCP TG mice (Fig. 4d). These data indicate that iNOS is necessary for the stimulation of respiration by VCP.

### iNOS inhibition prevents VCP-induced increase in mitochondrial respiration in isolated cardiomyocyte *in vitro*

To further determine whether the enhancement of mitochondrial respiration observed in VCP TG mice originated directly from cardiomyocytes, rat neonatal cardiac myocytes (RNCMs) were transfected with adenoviruses harboring the full-length VCP sequence (Ad-VCP) and compared to the Ad-β-Gal control (Ad-β-Gal). iNOS activity was increased by 2-fold upon the overexpression of VCP versus β-Gal. Addition of iNOS inhibitor 1400 W abolished VCP-mediated activation of iNOS (Fig. 4g). Consistent with the change in VCP TG mice, compared to Ad-β-Gal, RNCMs treated with Ad-VCP exhibited a 2.2-fold increase in oxygen consumption rates (OCR), which represents an index of mitochondrial respiration capacity. This effect was abolished by addition of the iNOS inhibitor, 1400 W (Fig. 4h). Therefore, these results in cultured cardiomyocytes offer further support to the results obtained from genetically-modified mice showing that the effect of VCP on mitochondrial respiration is dependent upon increased iNOS activity.

### Over-expression of VCP prevents mPTP opening

**Figure 5:**
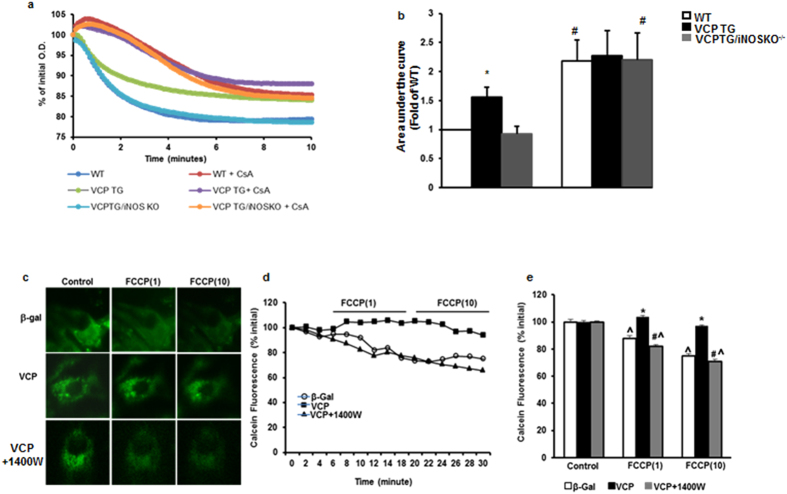
Effect of VCP overexpression on mPTP opening. (**a**) Representative examples of calcium-loaded swelling assay with or without the presence of Cyclosporine (CsA). (**b)** Quantitative representation of panels a. n = 5 per group *p < 0.05 vs. WT; ^#^p < 0.05 vs. VCP *TG.* (**c**) Representative examples of mitochondrial calcein fluorescence after addition of FCCP at the concentration of 1 μmol/L (1) or 10 μmol/L (10) in RNCMs. (**d)** Representative tracing curve of calcein fluorescence over time in the same groups. (**e)** Quantitative representation of panels d. n = 11 to 22/group. Data are the mean ± SEM. ^p < 0.05 vs. corresponding control, *p < 0.01 vs. Ad-β-Gal, ^#^p < 0.01 vs. Ad-VCP.

In order to further investigate the protective role of VCP in IR, we next examined its potential effect on the mPTP opening, which is a critical mechanism to promote cell damage during IR, particularly at the early stage of reperfusion^3^. Mitochondria were isolated from the heart of WT, VCP TG and VCP TG/iNOS KO^−/−^ mice. mPTP opening was induced by high concentration of calcium (600 μM CaCl_2_) and determined by the calcium–overload swelling assay. As showed in Fig. 5a and b, compared to the WT, the mitochondria isolated from the heart of VCP TG mice exhibits a significantly less decrease in absorbance at 540 nm upon the addition of CaCl_2_, indicating the inhibition of calcium induced mPTP opening. This protection was abolished by the deletion of iNOS in VCP TG/iNOS KO^−/−^ mice (Fig. 5b). Furthermore, addition of cyclosporine A (CsA), a known mPTP inhibitor, prevented the mPTP opening in all the three groups and eliminated the difference between WT and VCP TG (Fig. 5a and b). These data further support that VCP protects the mitochondria from the calcium-load induced mPTP opening.

mPTP opening was also determined in isolated cardiomyocytes by a reduction in mitochondrial calcein fluorescence signal after addition of FCCP, which is an uncoupler of the mitochondrial respiratory chain, at the concentration of 1 μmol/L or 10 μmol/L in myocytes treated for 48 hours with Ad-β-Gal and Ad-VCP with or without the iNOS inhibitor 1400 W (Fig. 5c). Calcein fluorescence signaling was traced over time after addition of FCCP (Fig. 5d). Compared to the untreated control, the calcein fluorescence signal in Ad-β-Gal-treated RNCMs declined upon FCCP treatment (p < 0.05), reflecting mPTP opening. RNCMs treated with Ad-VCP preserved the same calcein fluorescence signal as control cells upon FCCP stimulation, indicating that VCP prevented the induced mPTP opening in RNCMs. This prevention conferred by VCP was abolished by the addition of the iNOS inhibitor 1400 W (Fig. 5e). Therefore, increased expression of iNOS by VCP is necessary for the reduction in mPTP pore opening in cardiomyocytes.

## Discussion

**Figure 6:**
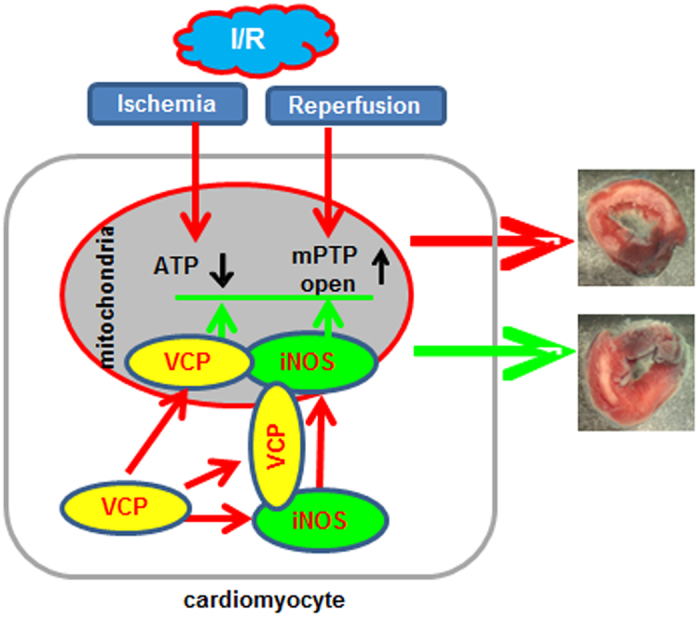
Summary of the findings. A schematic presentation of VCP action showing the potential mechanism of action of VCP.

As presented in Fig. 6, our results demonstrate that over-expression of VCP in a genetically-modified mouse model *in vivo* provides cardiac protection in ischemic heart equivalent to that provided by the SWOP^16^,^17^; This effect is linked to a preservation of the capacity of mitochondrial respiration and a prevention of mPTP opening under the stress; The mechanistic link between VCP-mediated cardioprotection and preservation of mitochondrial function under the cardiac stress is an increased expression and activity of iNOS. Taken together, these results demonstrate that VCP is a novel agonist of iNOS-mediated mechanisms of cardioprotection against ischemia.

Very little information is available about the function of VCP in the heart, at the opposite of other tissues. VCP is a member of the type I AAA (ATPases associated with various cellular activities). By interacting with several sets of adaptor proteins^19^, VCP is involved in a variety of cellular pathways that are essential for cell homeostasis, such as cell cycle control, transcriptional regulation, apoptosis, protein degradation, and cellular stress response^20^,^21^,^22^,^23^. Despite this information gained from other tissues, the physiology of VCP in the heart remains largely unknown. We first identified VCP in the heart when we showed that it acts as a novel downstream effector of the cardioprotective signaling mechanism conferred by Hsp22^15^. Our initial studies *in vitro* showed that VCP represents the link between Hsp22-mediated activation of Akt and nuclear factor-kappa B (NF-ĸB)-induced expression of iNOS in cardiac myocytes, thereby playing a central role in the mechanisms of cardiac cell survival promoted by Hsp22. We also showed that overexpression of VCP protects cardiomyocytes against apoptosis^15^. Based upon this evidence obtained *in vitro*, we tested the physiological relevance of our findings *in vivo* by generating a cardiac-specific VCP TG mouse.

In the present study, we identified for the first time a specific physiological function of VCP in the mammalian heart, in both normal and stress conditions. Our results show that no change was observed in TG mouse compared with WT in baseline conditions when considering heart mass, ventricular structure, contractile function, and the size and viability of cardiomyocytes. These data demonstrate that chronic stimulation of VCP at this overexpression level has no toxic effects on the heart tissue. Thus, the VCP TG mouse provides an original biological tool for the investigation of the mechanisms underlying VCP-mediated actions on cardiac stress.

A major observation of the present study is that overexpression of VCP protects the heart against IR-induced damage. Acute MI remains one of the leading causes of morbidity, mortality and disability worldwide^1^. Early and successful reperfusion is the most effective strategy for reducing the IS and for improving the clinical outcome. However, the process of restoring blood flow to the ischemic myocardium can create injury itself, for which no effective therapy is currently available^5^,^24^. IPC is well recognized as the most powerful endogenous mechanism of cardioprotection, however the clinical translation of IPC remains limited because of the problematic requirement of pre-emptive ischemic episodes^16^,^17^,^24^,^25^. Our results defined VCP as a new and powerful mediator of cardiac protection during IR, demonstrating that VCP may represent a novel therapeutic avenue for the prevention of MI.

Cardiomyocyte death induced by IR is the major cause of myocardial infarct and loss of cardiac function. VCP has been identified as an apoptosis regulator in other mammalian cells and in yeast^26^,^27^,^28^, Recent studies have identified that loss of VCP activity due to mutations is linked to cell death in different human diseases, including a variety of neurodegenerative diseases, such as Alzheimer’s disease, Pakinson’s disease and amyotrophic lateral sclerosis^26^,^29^,^30^. VCP has also been shown to be associated with cell survival of cancer cells^31^,^32^,^33^,^34^. We demonstrated previously the protective role of VCP against apoptosis in mammalian cardiomyocytes *in vitro*^15^. However, whether this anti-apoptotic effect of VCP protects the heart against ischemia-induced cell death remained unclear. Our data further show that overexpression of VCP by 3.5-fold is sufficient to provide prophylactic cardioprotection against cell death in the heart under ischemic stress.

Another important finding in the present study is a novel role for VCP in mitochondria of the mammalian heart. Previous mechanistic studies on VCP-mediated cell survival focus on its involvement in endoplasmic reticulum (ER) stress-triggered apoptotic pathway^35^,^36^. VCP deficiency is associated with decreased mitochondrial membrane potential in human dopaminergic neuroblastoma cell line^37^,^38^,^39^. It has been shown that mutations or deficiency of VCP cause profound mitochondrial dysfunction which results in a significant reduction of ATP synthesis, making neuronal cells more vulnerable to ischemia and cell death^37^,^38^,^39^. However, the potential role of VCP in cardiac mitochondria has not been tested. We first showed that, in a mouse model, VCP exhibits a preferred accumulation in cardiac mitochondria as compared to other sub-cellular fractions, which highlights the possibility of an important mitochondrial function for VCP under stress conditions. This is supported by our observation that VCP TG mice exhibit a significant elevation of the capacity of mitochondrial respiration in the state 3, with no change in states 2 and 4. State 3 is defined as ADP-stimulated respiration. When ADP binds ATP synthase, protons are driven into the matrix from the outside of the inner membrane. The energy released by this proton flux directly drives ATP synthesis. Therefore, an increase in state 3 respiration indicates an increased capacity of ATP synthesis^6^, while states 2 and 4 are viewed as the steady states of mitochondrial respiration. Therefore, an increase in RCR, which reflects the state 3/state 4 ratio, is an excellent indicator of improved respiratory activity and efficiency^6^. Additionally, the increase of oxygen consumption was blocked by the inhibitor of cytochrome oxidase, KCN, further supporting the enhancement of mitochondrial respiratory chain by VCP. These data together indicates that overexpression of VCP promotes mitochondrial respiration efficiency, which enhances cellular resistance to oxidative damage, and thus may act as a mechanism of the prevention of cardiomyocyte death during ischemia^6^.

There is also increasing evidence that mPTP opening plays a central role in mediating both the necrotic and apoptotic components of IR injury particularly at the onset of reperfusion^3^,^12^,^40^,^41^. Importantly, it has been shown that IPC attenuates IR-induced opening of the mPTP^12^. Recent studies also demonstrated that the use of mPTP opening inhibitors, such as cyclosporin A, reduces IS in animal models of acute IR injury^41^. Despite intensive investigation, the molecular identity of the mPTP remains undefined. Our findings showed that overexpression of VCP reduced mPTP opening, indicating that VCP targets an important mitochondrial regulator of cardioprotection against reperfusion injury during IR.

Our next observation is that VCP regulates mitochondrial function in an iNOS-dependent manner. The effect of VCP on mitochondria is related to iNOS for the following reasons: (1) VCP induces the expression of iNOS not only in cardiac myocytes *in vitro*^15^ but also in the heart of VCP TG mice *in vivo*. (2) The cytoprotection conferred by VCP was abolished by the addition of a selective iNOS inhibitor^15^. (3) iNOS is necessary for Hsp22-related mitochondrial regulation and cytoprotection^18^. To further investigate if the effect of VCP on mitochondrial function is mediated through the increase of iNOS expression, both pharmacological and genetic approaches were used. We first generated a bigenic mouse in which iNOS was deleted from the VCP TG mouse. This model confirms that enhanced mitochondrial respiration by VCP overexpression in the mouse heart was abolished by the deletion of iNOS. Importantly, VCP exhibited no stimulatory effect on another NOS isoform, such as eNOS. In addition, we also showed in isolated RNCMs that VCP overexpression results in stimulation of mitochondrial respiration, an effect which was eliminated upon treatment with the iNOS inhibitor 1400 W. Although the precise mechanism of VCP on mitochondrial function is largely unknown, our results imply that iNOS is a crucial mediator of such effect. Furthermore, it has been shown previously that mitochondria extracted from iNOS TG mouse hearts subjected to IR have a decreased mPTP opening compared to WT^42^. We demonstrated that overexpressing VCP decreased the rate of mPTP opening, and that such effect was completely abolished by the deletion or inhibition of iNOS, further indicating that iNOS is the critical mediator of the effect of VCP in the inhibition of mPTP opening.

Although the role of iNOS in cardioprotection has been firmly established, the effect of NO in the mitochondria has been controversial. Indeed, the detrimental effect of NOS on mitochondrial respiration were observed in several conditions related to excessive formation of NO^43^,^44^, such as during hypoxia^45^,^46^,^47^ or under treatment with exogenous NO stimulators^48^. However, our data clearly show that the moderate increase of endogenous iNOS by 2- to 3-fold, which reproduces the extent of overexpression during SWOP, provides cardiac protection during IR injury and promotes mitochondrial respiration. These data are also consistent with our previous observation in a Hsp22 TG mouse model^18^,^49^ as well as in ischemic preconditioning^50^. In addition, different conclusions were made when comparing the increased iNOS expression originating from resident inflammatory cells^51^ or from circulatory blood cells^52^ compared to the iNOS generated by cardiac myocytes^52^. Also, we recently showed that it, instead of the total cellular iNOS content; it is rather the abundance of iNOS in the mitochondria that is crucial for Hsp22-mediated stimulation of oxidative phosphorylation^18^. Accordingly, the present study shows that iNOS has a preferred location in mitochondria of VCP TG mouse heart, which further supports the concept that stimulation of endogenous iNOS expression and its subsequent translocation to the mitochondria in cardiac myocytes is crucial for its effects on both mitochondrial respiration and cardioprotection. This may also explain the divergent results observed in previous studies in which NO production originated from “non-myocyte” iNOS^47^,^52^ or from exogenous NO donors^48^. The cellular origin and subcellular distribution of iNOS are likely major determinants of its beneficial effect against ischemic injury and mitochondrial respiration. The VCP TG mouse that we generated provides a biological tool for the further study of the mechanisms underlying endogenous iNOS mediated protection.

In summary, the data presented in this study provide the following novel information in the heart *in vivo*: (1) Cardiac-specific overexpression of VCP in a TG mouse did not change the overall physiological and morphological characteristics of the mouse in baseline conditions. (2) The VCP TG mouse provides a significant reduction in IS during IR injury, which is quantitatively comparable to that observed in IPC. (3) Overexpression of VCP in the heart *in vivo* leads to an increase in endogenous iNOS expression and activity. (4) Increased iNOS by VCP shows a preferential localization to the mitochondria in TG mouse heart. (5) VCP overexpression leads to an increase in mitochondrial respiratory capacity and an inhibition of the rate of mPTP opening, which are dependent upon iNOS expression and activity. Altogether, these data further highlight a novel cardioprotective mechanism for VCP *in vivo*. Therefore, pre-emptive activation of VCP and its downstream targets may represent a novel approach to prevent ischemic damage in the heart at risk of suffering from subsequent ischemic stress.

## Methods

### Animal Models

#### Generation of the VCP TG mouse

A construct harboring a 2.4 Kb coding sequence of VCP was generated, in which the transgene expression was under the control of the cardiac-specific promoter of the α-myosin heavy chain (αMHC). The plasmid was digested with Bam HI, and introduced by pronuclear microinjection in zygotes of FVB mice, as described previously^53^. Positive mice were mated with wild type, and their offspring were screened.

#### Generation of the VCP TG/iNOS KO bigenic mouse

VCP TG mice (FVB) were cross mated with a homozygous iNOS KO^−/−^ (C57BL/6, Jackson Laboratory, stock Number: 002609). After 5 generations of background clearing, F1 generation (VCP TG/iNOS KO^+/−^ and WT/iNOS KO^+/−^) (FVB) mice were mated together to generate a VCP TG/iNOS KO^−/−^ homozygote (FVB). Litter-matched VCP TG (VCP TG/iNOS^+/+^) (FVB) and WT (WT/iNOS^+/+^) (FVB) were used as controls.

Three to four month-old mice, both male and female, were used for this study. Animals were euthanized with 100 mg/kg pentobarbital. The investigation conforms to the Guide for the Care and Use of Laboratory Animals published by the US National Institutes of Health (Publication No. 85-23, revised 2011), and with the approval of the IACUC committee at the local institutions (Rutgers, The State University of New Jersey, and Loma Linda University).

### Culture of RNCMs

RNCMs were prepared from Sprague-Dawley rat pups (Charles River Laboratories, Wilmington, MA) as described previously^15^,^18^,^49^,^54^. Recombinant adenoviruses (Ad) harboring the full-length VCP sequence (Ad-VCP) or β-Gal control (Ad-β-Gal) was generated with the AdEasy XL adenoviral vector system (Agilent, Santa Clara, CA). Myocytes were infected with Ad-VCP and Ad-β-Gal for 48 hours after 24 hours of serum-free starvation. Inhibition of iNOS was initiated 24 hours before the collection of the cells upon addition of 100 μmol/L 1400 W (Sigma-Aldrich, St. Louis, MO)^15^,^18^.

### Echocardiography

Cardiac function and LV structure were measured in the VCP TG mice and WT littermates by two-dimensional echocardiography as described previously^49^. After determination of body weight, mice were anesthetized with 2% isoflurane (JD Medical, AZ). Echocardiography was performed using a Logiq E vet with a 13-MHz probe (12L-RS). Heart rate, LVEDD and LVESD, wall thickness, and contractility (EF and FS) were measured.

### Surgical procedure

IR was performed on both VCP TG and WT mice and the infarct area and the area at risk were measured as described previously^55^. Briefly, mice were anesthetized with ketamine 65 mg/kg, xylazine 1.2 mg/kg, and acepromazine 2.17 mg/kg intraperitoneally and ventilated via tracheal intubation connected to a rodent ventilator with a tidal volume of 0.2 ml and a respiratory rate of 110 breaths per minute. The heart was exposed through a thoracotomy and the left anterior descending (LAD) artery was located and was occluded for a period of 45 min to induce ischemia followed by 24 hours of reperfusion. At the end of each experiment, the LAD was re-ligated to determine the area at risk by low-pressure retrograde injection of 0.5 mg/ml Alcian blue through the aorta. The infarct size was measured following incubation in 1% Triphenyl Tetrazolium Chloride (TTC) at 37 °C for 15 min. Image-Pro software was used to measure and analyze of the infarct area and the area at risk from each section.

### Histopathology

Hearts form both VCP TG and WT were collected and weighed. The LV/BW, LV/TL and LW/TL ratios were measured. Heart samples were fixed in 10% formalin and cut into 7-μm-thick sections. Collagen accumulation, myocyte cross-sectional area and myocyte apoptosis were measured as described previously^49^.

### qPCR and Western blotting

mRNA was extracted from mouse heart tissues and qPCR was performed as described previously^49^,^54^. Protein extraction and subcellular fractions were performed as described previously^49^,^54^. Targeted proteins including VCP, eNOS and iNOS were detected by western blotting as previously described^15^,^18^. The primary antibodies used for western blotting were anti-VCP (catalog no. 2648S, rabbit, dilution of 1:1000, Cell Signaling Technology), anti-iNOS (catalog no. ab136918, rabbit, dilution of 1:1000, Abcam), and anti-eNOS (catalog no. Sc-654, rabbit, dilution of 1:1000, Santa Cruz Biotechnology). Anti-GAPDH (Catalog no. ab9484, mouse, dilution of 1:10,000, Abcam) and anti- voltage-dependent anion-selective channel protein 1(VDAC) (Catalog no. 4866, rabbit, dilution of 1:5,000, Cell Signaling Technology) were used as loading controls.

### iNOS activity assay

iNOS activity was measured with the Nitric Oxide (NO) Assay Kit (Oxford Biomedical Research, Oxford, UK) as described previously^18^.

### Mitochondrial isolation

Mitochondria were isolated from mouse heart as described previously^15^,^18^,^49^. Briefly, sub-cellular fractions were prepared by differential centrifugation of tissue homogenized manually in a Dounce homogenizer using hypotonic buffer. After an initial spin at 100 g (5 minutes) to discard the cellular debris and unbroken cells, the nuclear fraction was pelleted at low-speed centrifugation (500 g, 10 minutes). The supernatant was further centrifuged (10,000 g, 10 minutes) to pellet the mitochondrial fraction. The resulting supernatant was ultra-centrifuged (100,000 g, 90 minutes) to obtain the cytosolic fraction (supernatant) and a microsomal fraction (pellet). Pellets were washed 3 times with 1x phosphate-buffered saline and re-suspended in RIPA buffer (150 mM NaCl, 1% NP40, 0.5% deoxycholate, 0.1% SDS, 50 mM Tris (pH 8.0))^15^,^18^,^49^. The purification of the isolated mitochondria from heart tissue was verified by western blotting, using glyceraldehyde 3-phosphate dehydrogenase (GAPDH, cytosol), histone 1 (nucleus), and voltage-dependent anion channel (VDAC, mitochondria) as described previously^15^,^18^,^49^.

### Oxygen consumption assay

Oxygen consumption of isolated mitochondria was measured at 30 °C with a Clark-type electrode fitted to a water-jacketed reaction chamber of 0.5 mL. Mitochondria (0.3–0.4 mg/mL) were isolated from mouse heart and incubated in a respiration buffer containing 100 mmol/L KCl, 50 mmol/L sucrose, 10 mmol/L HEPES, 5 mmol/L KH_2_PO_4_, pH 7.4. The following parameters of mitochondrial respiration were evaluated^49^,^56^: 1. Substrate-dependent respiration rate (state 2 and state 4 with 5 mmol/L pyruvate or 10 mmol/l glutamate/5 mmol/L malate) in the absence of exogenous ADP; 2. ADP-stimulated respiration rate (state 3) in presence of 250 mmol/L ADP; *3.* Oxygen-consumption with the addition of following reagents: 5 mmol/L succinate in the absence or in the presence of rotenone (2 μmol/L); cytochrome C (4 μmol/L); KCN (50 μmol/L) and oligomycin (10 μmol/L); 4. Respiration uncoupling in presence of 0.2 mmol/L FCCP.

Oxygen consumption rates (OCR) were also measured in intact RNCMs with a Clark-type electrode as described previously^18^. Briefly, RNCMs infected with Ad-β-Gal and Ad-VCP were collected and suspended in extracellular buffer as described^18^,^57^. Glucose was used as a substrate. OCR was determined after addition of 6 μmol/L oligomycin or 5 μmol/ FCCP. The ratio of FCCP-stimulated to oligomycin-inhibited OCR in the myocytes was calculated^18^.

### Mitochondrial swelling assays

Mitochondria were isolated as described above. mPTP opening was induced by calcium loading via addition of 600 μM of CaCl_2_. The mitochondrial swelling was monitored by the decrease in light-scattering at 540 nm in a spectrophotometer (Thermo Scientific Multiskan GO) every 6 second for 10 minutes in the presence or absence of 10 μM Cyclosporin A^58^.

### mPTP opening assay in intact cardiomyocytes

mPTP opening was determined by analyzing the mitochondrial calcein leak as previously described^59^,^60^. Briefly, RMCMs were loaded with 1 μmol/L calcein-AM and 1 mmol/L CoCl2 for 30 min at room temperature, and calcein fluorescence quenching was measured. mPTP opening was evaluated after addition of 1 μmol/L or 10 μmol/L FCCP, which uncouples the mitochondrial respiratory chain, and depolarizes mitochondrial membrane potential. Opening of mPTP promotes mitochondrial calcein quenching. During the whole experiments, the loaded cells were excited at 470 nm and the emitted light was collected at 510 nm. The mPTP opening was indicated by a reduction in mitochondrial calcein fluorescence signal.

### Statistical analysis

Results are the mean ± SEM for the number of samples indicated in the figure legends. A one-way ANOVA was used, and a Student-Newman-Keuls post hoc correction was applied for multi-group comparisons. A value of p < 0.05 was considered significant.

## Additional Information

**How to cite this article**: Lizano, P. *et al*. The valosin-containing protein is a novel mediator of mitochondrial respiration and cell survival in the heart *in vivo. Sci. Rep.* **7**, 46324; doi: 10.1038/srep46324 (2017).

**Publisher's note:** Springer Nature remains neutral with regard to jurisdictional claims in published maps and institutional affiliations.

## Figures and Tables

**Table 1:** Physiological and morphological characteristics of VCP TG mice compared to WT littermates.

	WT (n = 6)	TG (n = 6)
Body weight (g)	24.4 ± 0.4	24.6 ± 0.9
LV/body weight (mg/g)	3.2 ± 0.1	3.3 ± 0.2
LV/tibial length (mg/mm)	3.6 ± 0.1	3.8 ± 0.1
Ling weight/tibial length (mg/mm)	8.0 ± 0.4	8.0 ± 0.5
LV Ejection fraction (%)	71.9 ± 0.9	71.2 ± 1.2
LV Fractional shortening (%)	34.5 ± 0.7	34.0 ± 0.9
Heart rate (bpm)	481 ± 4.7	486 ± 5.7
LV End-diastolic septal wall thickness (mm)	0.72 ± 0.02	0.72 ± 0.01
LV End-diastolic posterior wall thickness (mm)	0.65 ± 0.01	0.66 ± 0.01
LV End-diastolic diameter (mm)	3.1 ± 0.02	3.1 ± 0.01
LV End-systolic diameter (mm)	2.0 ± 0.02	2.1 ± 0.02
Myocyte cross-sectional area (µm^2^)	328 ± 15	329 ± 16
Apoptosis positive cells (‰ Nuclei)	0.2 ± 0.07	0.2 ± 0.03
Collagen (% surface)	3.5 ± 0.9	3.4 ± 0.7
